# IITR00803: a benzoxazole-nitrothiophene small molecule with broad-spectrum antibacterial potential

**DOI:** 10.1128/spectrum.01144-25

**Published:** 2025-08-12

**Authors:** Rinki Gupta, Amit Gaurav, Ranjana Pathania

**Affiliations:** 1 Department of Biosciences and Bioengineering, Indian Institute of Technology Roorkee 30112, Roorkee, Uttarakhand, India; University of Manitoba, Winnipeg, Manitoba, Canada

**Keywords:** antibacterial small molecule, antibiotic resistance, drug discovery, nitrothiophene, DNA damage

## Abstract

The drug discovery of novel antibacterials is pivotal to combat the ever-growing challenge of antibiotic resistance. The current antibiotics in clinics target the essential pathways in bacteria. However, drug efflux among other resistance mechanisms remains a persistent problem, leading to reduced efficacy of antibiotics. In the present study, we report the discovery of a novel small molecule, IITR00803, with antibacterial properties. IITR00803 demonstrated broad-spectrum antibacterial activity with potent inhibition of enteric pathogens. The small molecule contains a benzoxazole group and a nitrothiophene moiety in its chemical structure. The nitrothiophene-based compounds are prone to efflux by AcrAB-TolC efflux pumps. Despite containing the nitrothiophene group, IITR00803 did not demonstrate efflux liability, thereby demonstrating a broad antibacterial spectrum. The small molecule depicted bactericidal activity against *Salmonella enterica* serotype Typhimurium with a post-antibiotic effect of ~1.51 h. The mode of action studies revealed the membrane potential perturbing properties of IITR00803 against *S. enterica* serovar Typhimurium and *Escherichia coli,* whereas DNA-damaging activity was observed against *E. coli*. Resistance development studies on IITR00803 in laboratory growth media showed that *S. enterica* serovar Typhimurium could not develop stable resistance against this molecule under the tested conditions. Furthermore, IITR00803 exhibited non-toxic potential against eukaryotic cells and the model organism *Caenorhabditis elegans*. The compound also depicted efficacy against the *C. elegans* infection model of *S. enterica* serovar Typhimurium. Collectively, our results show that IITR00803 is a broad-spectrum antibacterial small molecule that alters membrane potential and exhibits *in vitro* and *in vivo* safety and efficacy.

**IMPORTANCE:**

Antimicrobial resistance is a growing global health threat, necessitating the urgent development of novel antibacterial agents. *Salmonella* infections, particularly those originated by non-typhoidal serovars such as *Salmonella enterica* Typhimurium and *S. enterica* Enteritidis, contribute significantly to morbidity and mortality worldwide. These foodborne pathogens cause severe gastroenteritis and spread through poor sanitation and food safety measures, leading to millions of infections each year. The emergence of multidrug-resistant *Salmonella* strains has further complicated treatment by limiting the therapeutic options. This crisis highlights the need for the discovery of new therapeutic options that impede the resistance development of bacterial pathogens and demonstrate the potential to overcome existing antibiotic resistance mechanisms.

## INTRODUCTION

Antimicrobial resistance (AMR) poses a significant health threat and is responsible for escalating mortality rates. AMR occurs when microbes evolve and develop mechanisms to resist the efficacy of drugs that once inhibited their growth (1). The World Health Organization (WHO) has identified foodborne pathogens as a major public health concern and classified foodborne diseases as a significant global burden, with *Salmonella* being one of the leading causes of bacterial foodborne infections. In 2015, the WHO estimated that 31 foodborne hazards were responsible for 600 million cases of foodborne diseases globally, leading to 420,000 deaths (2). Innovative therapeutics targeting enteric bacterial pathogens such as *Salmonella* can help mitigate the global burden of foodborne illnesses and AMR-related challenges. The increasing prevalence of AMR necessitates a multifaceted approach to combat its spread. This includes the discovery of novel antimicrobial agents, in-depth exploration of their mechanisms of action, and resistance development pathways in bacterial pathogens to maintain their efficacy in clinics. An important aspect of antibacterial drug discovery involves the utilization of small-molecule compounds as antibacterials (3). The small molecules prove to be useful as antibacterial compounds owing to their low cost, ease of use, and modification. The low molecular weight and varied chemical structures of small molecules render them appropriate for engineering to improve their physicochemical properties.

In recent years, nitrothiophenes have emerged as a promising class of nitro-heterocyclic compounds with antibacterial properties (4–7). These molecules have shown potential against a range of Gram-positive and Gram-negative pathogens. However, a major limitation of nitrothiophenes is their susceptibility to efflux via the AcrAB-TolC efflux pump, a multidrug resistance (MDR) transporter, thereby reducing their intracellular concentrations and antibacterial effectiveness (8). This efflux liability significantly limits the therapeutic potential of nitrothiophenes, prompting efforts to design and synthesize derivatives with reduced efflux predisposition. Furthermore, nitroaromatic compounds like nitrofurans are prodrugs that are activated in the presence of nitroreductases (9).

In the present study, we report the discovery of a novel nitrothiophene-based small molecule, IITR00803, which demonstrates broad-spectrum antibacterial activity and is notably refractory to efflux mechanisms. The small molecule exhibited potent antibacterial activity against enteric pathogens, including clinical isolates, and displayed bactericidal activity. IITR00803 showed non-toxic potential against eukaryotic cells and the model organism *Caenorhabditis elegans*. The molecule further depicted efficacy against the *C. elegans* infection model of *Salmonella enterica* serovar Typhimurium and prevented stable resistance development. A detailed investigation of the mode of action revealed that IITR00803 alters membrane potential in both *Escherichia coli* and *S. enterica* serovar Typhimurium, whereas it also demonstrates DNA-damaging activity in *E. coli*. Our findings report IITR00803 as a bactericidal compound that exhibits *in vivo* safety and efficacy in the *C. elegans* model.

## RESULTS

### IITR00803 is a broad-spectrum, antibacterial small-molecule

**Fig 1:**
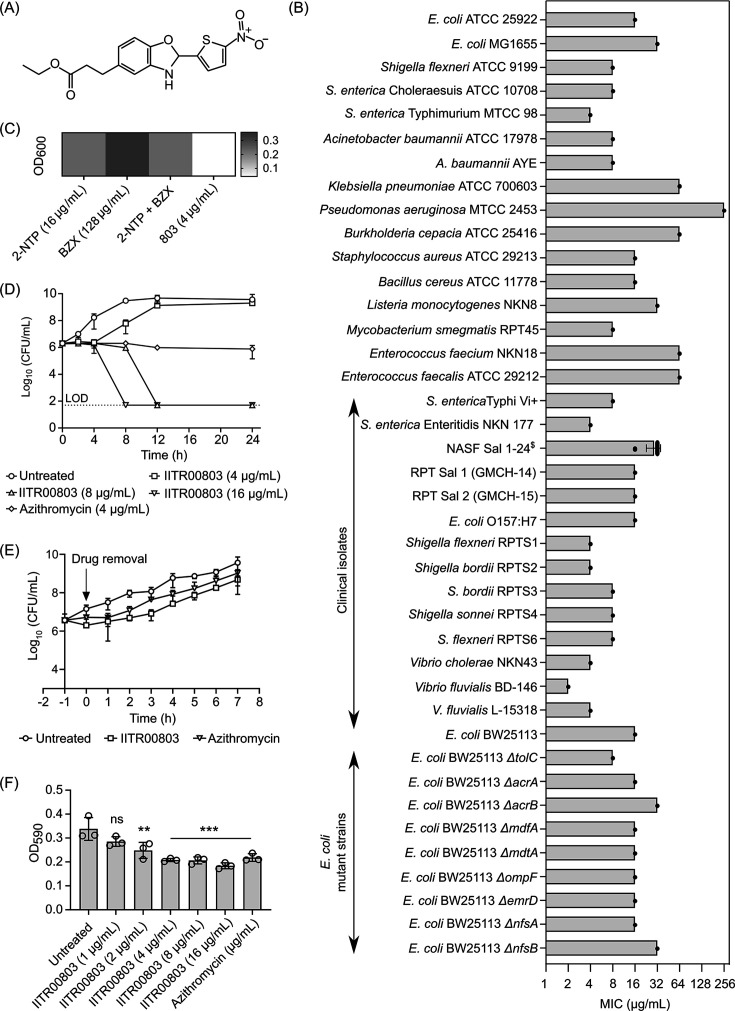
IITR00803 depicts favorable antibacterial properties against *S. enterica* serovar Typhimurium. (**A**) Chemical structure of IITR00803. (**B**) Bar graph representing MICs of IITR00803 against different bacterial strains. Each point represents the number of strains for each bacterium. For all other bacteria except NASF Sal^$^, only one point is shown; ^$^ denotes 24 different isolates of *Salmonella species*. (**C**) Heat map demonstrating bacterial growth inhibition profile in the presence of 2-NTP, BZX, and IITR00803. (**D**) Kill kinetics assay demonstrating the bactericidal nature of IITR00803. Azithromycin (4 µg/mL; 4× MIC) was used as a positive control. (**E**) Post-antibiotic effect of IITR00803. IITR00803 (16 µg/mL; 4× MIC) showed a post-antibiotic effect (PAE) of 1.51 h as compared to 1.02 h PAE of azithromycin (4 µg/mL). (**F**) Biofilm eradication assay representing anti-biofilm activity of IITR00803. Data (*n* = 3) are represented as Mean ± S.D. *P* values were determined by one-way analysis of variance (ANOVA) followed by Dunnett’s multiple comparison test. ***, *P* < 0.001; ns, non-significant.

IITR00803 was discovered as a lead antibacterial compound through a forward chemical genetic screening of ~11,000 small molecules against *E. coli* ATCC 25922, previously conducted in our laboratory (10). The chemical structure of IITR00803 comprises nitrothiophene and benzoxazole moieties (Fig. 1A). We examined the antibacterial efficacy of IITR00803 against a diverse panel of Gram-negative and Gram-positive bacterial pathogens. Our findings indicated that IITR00803 exhibits broad-spectrum antibacterial activity, with minimum inhibitory concentrations (MICs) ranging from 4 to 16 µg/mL against enteric pathogens such as *Salmonella* spp., *Shigella flexneri*, and *E. coli* (Fig. 1B). Given the potent anti-enteric activity of IITR00803, we further determined the MICs of IITR00803 against clinical isolates of enteric bacteria. The small molecule exhibited consistent antibacterial activity across all tested strains, with MICs ranging from 4 to 32 µg/mL against clinical isolates (Fig. 1B). These results highlight the clinical efficacy of IITR00803 against enteric pathogens. Furthermore, we compared the antibacterial activity of IITR00803 individually with 2-nitrothiophene (2-NTP) and benzoxazole (BZX) moieties which are present in the chemical structure of the small molecule (Fig. 1A). We found that IITR00803 depicts lower MICs against *E. coli* (16 µg/mL) and *S. enterica* serovar Typhimurium (4 µg/mL) as compared to both 2-NTP (>128 µg/mL for *E. coli* and 64 µg/mL for *S. enterica*) and BZX (>128 µg/mL for both strains) (Table S1). Moreover, to understand if the antibacterial killing effect of IITR00803 was due to the synergistic effect of these moieties, we performed a checkerboard assay in *S. enterica* serovar Typhimurium. We observed that these chemical moieties do not demonstrate synergy (Fig. 1C). On the contrary, the IITR00803 molecule, which contains both moieties, depicted bacterial killing at 4 µg/mL (Fig. 1C). These findings proved that IITR00803 does not depict enhanced antibacterial potential due to the synergy between 2-NTP and BZX but possesses this effect due to its unique structure.

### IITR00803 is not prone to efflux by the AcrAB-TolC efflux pump and is not a pro-drug

Nitrothiophenes are known to act as prodrugs, undergoing activation by bacterial nitroreductases (11). However, their antibacterial efficacy is often limited due to their susceptibility to efflux via the AcrB component of the Resistance-Nodulation-Division (RND) efflux pump (8). To test the efflux-prone nature of IITR00803, we evaluated the MICs of IITR00803 against various efflux pump deletion mutant strains of *E. coli* BW25113 (Fig. 1B). Evidently, IITR00803 showed no marked difference in MICs between wild-type and efflux-deficient strains (Fig. 1B). Additionally, although nitrothiophenes are typically activated by bacterial nitroreductases, our results showed that IITR00803 maintains similar MICs in wild-type *E. coli* BW25113, nitroreductase-deficient strains (Δ*nfsA* and Δ*nfsB*), and *E. coli* AG1 pCA24N *nfsA* overexpression strain (Fig. 1B; Table S2). This suggests that IITR00803 does not function as a prodrug and does not rely on nitroreductase-mediated activation for its antibacterial activity.

### IITR00803 is bactericidal in nature

The clinical importance and WHO-classified high-priority status of *Salmonella* and the potent antibacterial activity of IITR00803 against enteric pathogens prompted us to focus on assessing the pharmacodynamic properties of IITR00803 against *S. enterica* serovar Typhimurium. IITR00803 exhibited bactericidal activity in kill kinetics assay, reducing the bacterial load by over 3 Log_10_ CFU/mL at concentrations of 8 µg/mL (2× MIC) and 16 µg/mL (4× MIC) within 12 and 8 h, respectively (12) (Fig. 1D). These results indicated that the small molecule possesses bactericidal activity by reducing the bacterial burden to non-detectable colonies. Notably, IITR00803 outperformed azithromycin, an antibacterial agent commonly used to treat *Salmonella* infections, which depicted bacteriostatic activity at 4 µg/mL (4× MIC). Furthermore, we assessed the post-antibiotic effect (PAE) of IITR00803. PAE is an important pharmacodynamic parameter that helps in the assessment of bacterial growth suppression when the drug is removed or is present in sub-inhibitory concentrations, which helps in optimizing dosing regimens of any given antibiotic (13). IITR00803 displayed a PAE of approximately 1.51 h, which was about 30 min longer than that of azithromycin (~1.02 h) at their respective 4× MICs (Fig. 1E). Given that bacteria such as *Salmonella* form biofilms to enhance survival by providing protection against antibiotics, immune responses, and environmental stresses, we next evaluated the antibiofilm potential of IITR00803. Biofilm formation by *Salmonella* is of particular concern in the food industry, as this pathogen can colonize both food products and abiotic surfaces such as stainless steel (14). IITR00803 effectively eradicated preformed biofilms as well as prevented biofilm formation by *S. enterica* serovar Typhimurium under static conditions (Fig. 1F; Fig. S1). These results signify the bactericidal and anti-biofilm properties of IITR00803.

### IITR00803 alters cellular energetics and prevents resistance development

**Fig 2:**
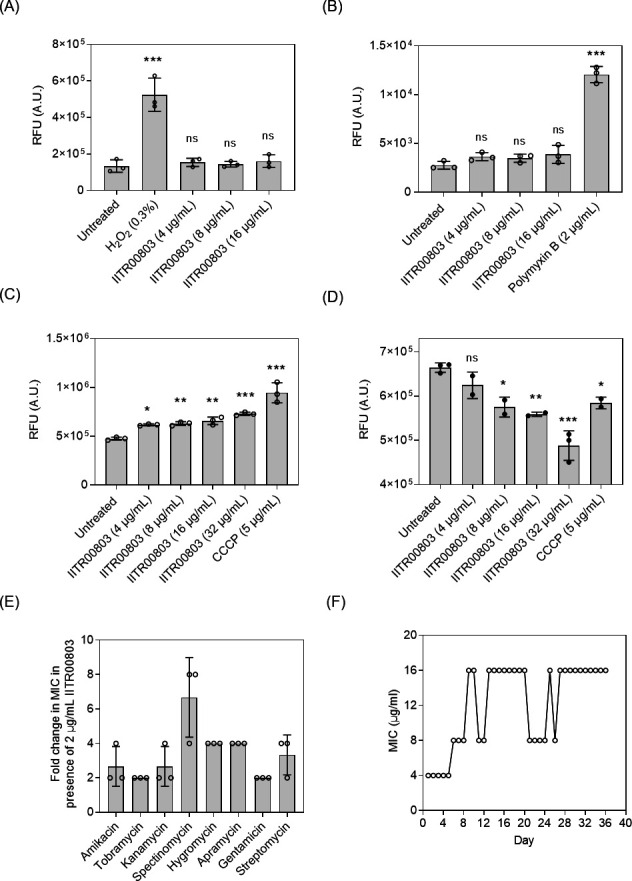
IITR00803 targets cellular energetics and prevents resistance development in *S. enterica* serovar Typhimurium. (**A**) Relative fluorescence of H_2_DCF-DA upon IITR00803 treatment. H_2_O_2_ was used as a positive control. (**B**) Relative fluorescence of Sytox Orange upon IITR00803 treatment. Polymyxin B was used as a positive control. (**C**) Relative fluorescence of DiBAC_4_(5) upon IITR00803 treatment. Carbonyl Cyanide m-Chlorophenyl Hydrazone (CCCP) was used as a positive control. (**D**) Relative fluorescence of resazurin showing NAD(P)H level after IITR00803 treatment. CCCP was used as a positive control. (**E**) Antibiotic potentiation assay reveals the change in MIC of aminoglycosides in the presence of 2 µg/mL of IITR00803. Data (*n* = 3) are represented as Mean ± S.D. *P* values were determined by one-way ANOVA followed by Dunnett’s multiple comparison test. *, *P* < 0.05; **, *P* < 0.01; ***, *P* < 0.001, ns represents non-significant. (**F**) Change in MIC of IITR00803 over 36 days of continuous passage.

Bactericidal antibiotics are generally known to induce bacterial cell death through the generation of reactive oxygen species (ROS), leading to membrane damage, lipid peroxidation, and DNA damage (15). To assess the ROS-generating potential of IITR00803, we checked ROS generation using the ROS-sensitive probe, H_2_DCF-DA (16). However, treatment with IITR00803 did not show a notable change in ROS production compared to the untreated control (Fig. 2A). To further investigate the mechanism of action, we evaluated the impact of IITR00803 on the bacterial membrane. We first investigated the effect of IITR00803 on membrane integrity using the Sytox Orange fluorescent probe. Sytox Orange is a cell-impermeable DNA-binding probe (17). A compromise in the membrane integrity results in more binding of the probe to DNA, leading to enhanced fluorescence, thereby depicting membrane damage. IITR00803-treated *S. enterica* serovar Typhimurium did not exhibit any substantial increase in fluorescence, indicating no membrane damage (Fig. 2B). This non-membrane-damaging effect of IITR00803 was further confirmed by scanning electron microscopy (SEM), which revealed intact bacterial cell membranes in IITR00803-treated and untreated *S. enterica* serovar Typhimurium (Fig. S2A). Furthermore, we examined the effect of IITR00803 on the membrane potential of *S. enterica* serovar Typhimurium using the DiBAC_4_(5) probe (18). Treatment with the molecule resulted in membrane depolarization, indicating disruption of membrane potential (Fig. 2C). Disruption of membrane potential is known to perturb bacterial cellular metabolism. Therefore, we assessed the NAD(P)H levels in bacterial cells after treatment with IITR00803 using resazurin indicator dye. Resazurin is a cell-permeable, blue-colored, and weak fluorescent dye that gets reduced to a pink and strongly fluorescent product, resofurin, in metabolically active cells (19). Our results revealed that IITR00803 treatment led to a reduction in NAD(P)H levels, as observed from the concentration-dependent reduction in the formation of resorufin (Fig. 2D). Membrane potential alteration is also known to enhance the efficacy of aminoglycoside antibiotics (20, 21). Consequently, we investigated the ability of IITR00803 to potentiate aminoglycoside activity by determining the MICs of several aminoglycoside antibiotics (amikacin, tobramycin, kanamycin, spectinomycin, hygromycin, apramycin, gentamicin, and streptomycin) in the presence of sub-inhibitory concentrations of the small molecule. The results demonstrated an enhanced antibacterial efficacy of aminoglycosides in the presence of IITR00803, reducing their MICs by twofold to eightfold (Fig. 2E; Table S3). Motivated by the aforementioned observation, we examined whether IITR00803 could also potentiate the activity of other antibiotics. We performed the same assay with representative antibiotics of other classes. We observed that sub-inhibitory concentrations of IITR00803 could also potentiate the activity of other antibiotics such as nitrofurantoin, furazolidone, and polymyxins by twofold to fourfold (Fig. S2B). The antibacterial agents targeting cellular bioenergetics have been shown to synergize with these antibiotics (22). The results depicted that IITR00803 acts by disrupting the bacterial membrane potential in *S. enterica* serovar Typhimurium and has no other damaging effects on the membrane. To determine whether the observed effects of IITR00803 were specific to *S. enterica* serovar Typhimurium or broadly applicable to other Gram-negative bacteria, we extended our analysis to *E. coli*. Consistent with our earlier findings, IITR00803 treatment in *E. coli* also resulted in membrane depolarization and potentiation of aminoglycosides without inducing ROS production or compromising membrane integrity (Fig. S3; Table S3). The absence of ROS generation and membrane damage suggests that the bactericidal activity of IITR00803 is mechanistically distinct from that of conventional bactericidal antibiotics, which often rely on oxidative stress to exert their effects. Instead, IITR00803 appears to exert its antibacterial action primarily through perturbation of cellular bioenergetics, as evidenced by membrane depolarization and a consequent reduction in intracellular NAD(P)H levels. This mode of action likely underlies its observed ability to potentiate the efficacy of aminoglycosides and other antibiotic classes. Furthermore, the consistent phenotype observed in both *S. enterica* serovar Typhimurium and *E. coli* suggests a conserved mechanism of action across Gram-negative bacteria, supporting its potential as a broad-spectrum antibacterial agent that targets bacterial energetics without inducing collateral oxidative or structural membrane damage. The broad-spectrum antibacterial activity, bactericidal activity, and ability to target cellular energetics of IITR00803 motivated us to investigate the bacterial resistance development potential against the small molecule. To assess this, we subjected *S. enterica* serovar Typhimurium cells to repeated exposure to IITR00803 at sub-inhibitory concentrations over a 36-day period. By the end of this duration, the bacterial cells exhibited a fourfold increase in MIC, rising from an initial 4 to 16 µg/mL (Fig. 2F). However, upon subsequent culturing in a drug-free medium, these cells reverted to their original susceptibility, with the MIC returning to 4 µg/mL. The observed transient increase in MIC during passage likely reflects a reversible adaptive mechanism that diminished once selective pressure was removed. Furthermore, this reversion indicates that IITR00803 does not induce stable resistance development in the *in vitro* tested laboratory conditions, thereby preventing the emergence of resistant mutants.

### IITR00803 depicts DNA-damaging potential in *E. coli*

**Fig 3:**
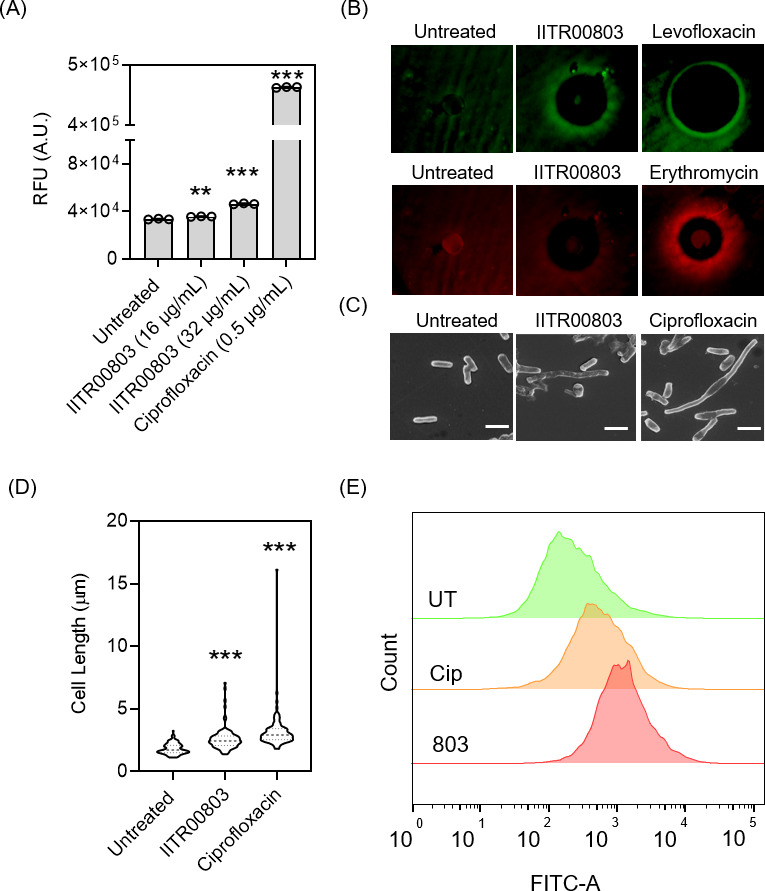
IITR00803 causes cell elongation and DNA damage in *E. coli*. (**A**) Biosensor assay representing *sulA* promoter expression in the presence of IITR00803. Ciprofloxacin treatment was used as a positive control. Data (*n* = 3) are represented as Mean ± S.D. *P* values were determined by one-way ANOVA followed by Dunnett’s multiple comparison test. *, *P* < 0.05; **, *P* < 0.01; ***, *P* < 0.001. (**B**) Agar plate images showing fluorescence signal of *E. coli* transformed with the pDualRep2 plasmid under different antibacterial stress conditions. Induction of the *SulA* promoter (pseudo green color) and TrpL2A attenuator (pseudo red color) indicates DNA and protein synthesis inhibition, respectively. Levofloxacin (DNA synthesis inhibitor) and erythromycin (protein synthesis inhibitor) were used as positive controls. (**C**) Scanning electron micrographs of *E. coli* cells showing cell elongation phenotype after treatment with IITR00803. Ciprofloxacin (1 µg/mL) was used as a positive control. Scale bars represent 2 µm. (**D**) Morphometric analysis of IITR00803-treated *E. coli* cells. Ciprofloxacin was used as a positive control. Each violin represents data points of approx 100 cells. The Kruskal-Wallis test was applied for statistical analysis. Major dotted lines represent the median, and minor dotted lines represent one-fourth and three-fourth quartiles. (**E**) TUNEL assay showing DNA damage upon IITR00803 treatment. Ciprofloxacin (1 µg/mL) was used as a positive control.

**Fig 4:**
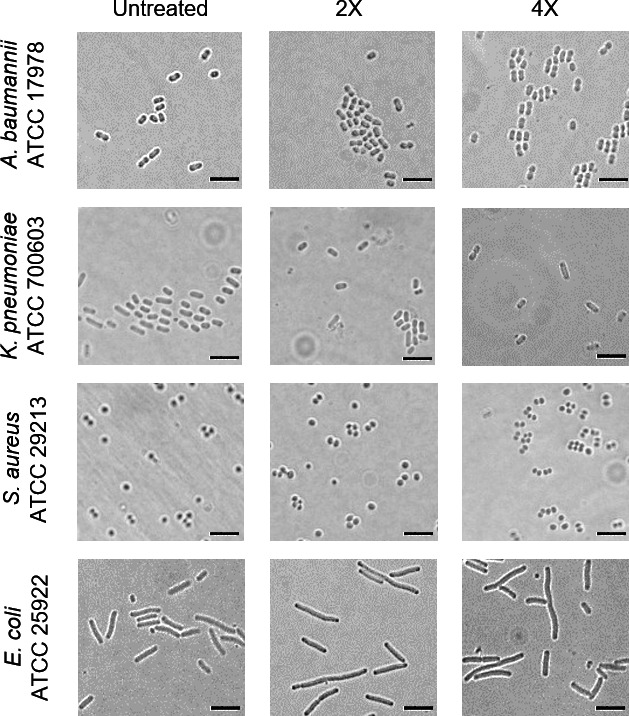
Microscopy images of bacterial cells treated with IITR00803. *A. baumannii* ATCC 17978, *K. pneumoniae* ATCC 700603, *S. aureus* ATCC 29213, and *E. coli* ATCC 25922 cells were treated with respective 2× and 4× MICs of IITR00803. The scale bar represents 5 µm.

To further investigate the effects of IITR00803 on other biomolecules such as DNA and protein synthesis machinery, we employed the pDualRep2 plasmid system (23). This plasmid construct contains *rfp* and *katushka2S* genes, which respond to DNA-damaging and ribosome-stalling agents, respectively. *rfp* is present under the *sulA* promoter, whose expression is induced maximally during DNA damage, whereas *katushka2S* is translationally fused downstream to tryptophan attenuator under a strong T5 constitutive promoter. When there is DNA damage in the cells, RFP produces red fluorescence, while Katushka2S produces far-red fluorescence when ribosomes are stalled by the translational inhibitors. We observed that *E. coli* cells treated with IITR00803 demonstrated a considerable enhancement of *sulA* expression as compared to untreated cells, whereas no considerable fluorescence was observed in the case of Katushka2S for translation inhibition (Fig. 3A and B). These results showed that IITR00803 might have a DNA-damaging effect in *E. coli*. DNA-targeting antibiotics such as quinolones and fluoroquinolones are known to cause cell elongation upon treatment (24, 25). This elongation phenotype has been associated with overexpression of *sulA,* which is a global SOS response gene (26). SulA binds to FtsZ and prevents cell division, leading to an elongated phenotype (27, 28). To evaluate whether IITR00803 causes cellular elongation, we treated *E. coli* cells with IITR00803, which resulted in noticeable cell elongation, as observed through SEM analysis (Fig. 3C). A morphometric analysis of treated versus untreated cells (*n* = 100–117) confirmed a substantial alteration in bacterial morphology (Fig. 3D). These results indicated that IITR00803 causes DNA damage in *E. coli* cells. To further validate these observations, we performed a Terminal Deoxynucleotidyl Transferase-Mediated dUTP Nick End Labeling (TUNEL) assay in *E. coli* due to the lack of observable phenotypic changes in the morphology of *S. enterica* serovar Typhimurium using ciprofloxacin as a positive control (29). We found that there was an increased Fluorescein Isothiocyanate (FITC) fluorescence in IITR00803-treated samples, which is indicative of DNA damage (Fig. 3E). These findings suggested that IITR00803 exerts a cell elongation phenotype in *E. coli* but not *S. enterica* serovar Typhimurium. Therefore, we further examined IITR00803 for its morphological impact on other bacterial pathogens, including *Acinetobacter baumannii* ATCC 17978, *Klebsiella pneumoniae* ATCC 700603, *Staphylococcus aureus* ATCC 29213, and *E. coli* ATCC 25922. These bacterial strains, except for *E. coli* ATCC 25922, did not depict any change in their morphology consistent with the results obtained for *S. enterica* serovar Typhimurium in terms of cell elongation phenotype (Fig. 4). These results showed that IITR00803 treatment does not exhibit a cell elongation phenotype on all bacterial pathogens tested. While *E. coli* exhibited morphological changes, other strains, including *S. enterica* serovar Typhimurium, *A. baumannii*, *K. pneumoniae*, and *S. aureus*, did not show such alterations, suggesting a strain-specific morphological response. However, our earlier observations revealed that IITR00803 disrupts membrane potential and alters cellular bioenergetics in both *E. coli* and *S. enterica* serovar Typhimurium in a similar manner. This suggests that while the core antibacterial mechanism of action of IITR00803 may be conserved, the phenotypic outcomes, such as changes in cell shape, could be influenced by intrinsic differences in the stress response and adaptive physiology of individual bacterial species.

### IITR00803 demonstrates *in vivo* safety and efficacy in *C. elegans*

**Fig 5:**
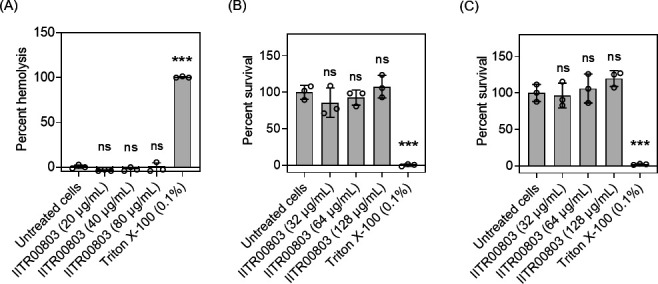
IITR00803 exhibits safety against eukaryotic cells. (**A**) Hemolysis assay reveals the non-hemolytic nature of IITR00803. (**B, C**) Cytotoxicity assay demonstrates the non-toxic nature of IITR00803 against eukaryotic cell lines—PMNs and PBMCs, respectively. Triton X-100 was used as a positive control. Data (*n* = 3) are represented as Mean ± S.D. *P* values were determined by one-way ANOVA followed by Dunnett’s multiple comparison test. ***, *P* < 0.001; ns, non-significant.

**Fig 6:**
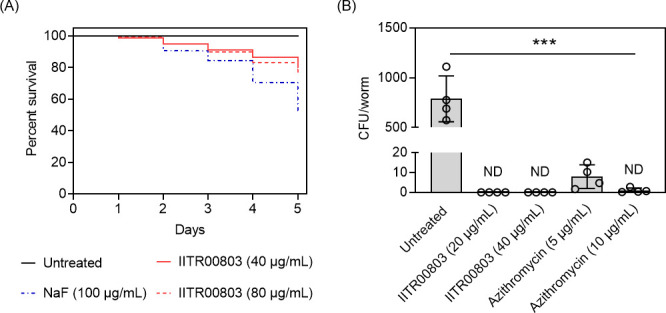
IITR00803 depicts *in vivo* safety and efficacy in *C. elegans*. (**A**) Kaplan-Meier survival curve showing *in vivo* toxicity in the *C. elegans* model. IITR00803 is non-toxic at concentrations up to 20× MICs, with a survival rate of 75%–80% over a period of 5 days. Sodium fluoride (NaF) was used as a positive control. (**B**) Bar graph representing efficacy of IITR00803 in *S. enterica* serovar Typhimurium-infected *C. elegans* model by substantially reducing the bacterial burden to non-detectable colonies within 12 h of treatment. Azithromycin was used as a positive control. ND denotes not detectable. Data (*n* = 4) are represented as Mean ± S.D. *P* values were determined by one-way ANOVA followed by Dunnett’s multiple comparison test. ***, *P* < 0.001; ns, non-significant.

The evaluation of the safety profile of antibacterial molecules is a critical factor in determining their therapeutic index (TI) and overall efficacy. The TI is defined as the ratio of the toxic dose to the effective dose (MIC), with a TI greater than 10 generally considered safe (30). To assess the safety of IITR00803, we conducted *in vitro* toxicity studies using freshly isolated human red blood cells, peripheral blood mononuclear cells (PBMCs), and polymorphonuclear cells (PMNs). IITR00803 demonstrated a non-hemolytic profile (Fig. 5A). Additionally, the compound exhibited no substantial cytotoxic effects on PBMCs and PMNs, achieving a TI greater than 32, as indicated by the lack of notable changes in cell viability compared to untreated controls (Fig. 5B and C). Furthermore, we assessed the *in vivo* toxicity and efficacy of IITR00803 using a *C. elegans* model. Toxicity studies in *C. elegans* correlate well with oral toxicity in rodents (31). The compound was found to be non-toxic to *C. elegans*, with 75%–80% survival rates observed after 5 days of exposure to concentrations equivalent to 20× MIC (80 µg/mL of IITR00803) (Fig. 6A). *In vivo* efficacy assessment of the compound on *S. enterica* serovar Typhimurium infected *C. elegans* indicated that IITR00803 was able to reduce the bacterial burden from worms within 12 h at 5× MICs leading to non-detectable colonies (Fig. 6B). These findings demonstrate that IITR00803 is a safe and effective therapeutic candidate for the treatment of *S. enterica* serovar Typhimurium infections, with promising *in vitro* and *in vivo* profiles.

## DISCUSSION

AMR is a growing global health crisis that poses a significant threat to public health, leading to prolonged illnesses, higher healthcare expenses, and a greater risk of spreading resistant infections (32). The discovery of novel antibacterial agents is important to combat AMR and ensure effective therapeutic options remain available for bacterial infections. Innovative antibacterials with distinct modes of action are needed to target MDR pathogens and overcome existing resistance pathways and cross-resistance. Our study reports the discovery of a novel antibacterial compound, IITR00803, which is a nitrothiophene-containing small molecule. Nitrothiophenes are broad-spectrum antibacterials and have been known to effectively target clinically relevant pathogens, including *E. coli*, *K. pneumoniae*, *S. flexneri*, *S. aureus,* and *Salmonella* spp. (8, 33, 34). These molecules contain a nitro group attached to an aromatic heterocyclic ring. The nitro group undergoes reduction by bacterial nitroreductases, which leads to oxidative stress, which in turn causes damage to DNA, membranes, and proteins, resulting in lethality. These multi-modes of action reduce the ability of bacteria to develop resistance, as quite a few mutations would be required to impede the action of these compounds. Despite their potential, nitrothiophenes face challenges related to bacterial efflux mechanisms, particularly by the AcrAB-TolC system in Gram-negative bacteria. However, efforts have been made to synthesize nitrothiophene derivatives with improved antibacterial efficacy (8, 33, 35, 36).

Our findings highlight the bactericidal and anti-biofilm properties of IITR00803, suggesting its potential as an effective alternative to traditional antibiotics for treating *Salmonella* infections. *S. enterica* is a clinically relevant human pathogen that is known to cause Salmonellosis, a significant concern in healthcare (37). The non-typhoidal serovars of *S. enterica*—Typhimurium (STM) and Enteritidis (SENT) have a broad-spectrum range of hosts and are the major causes of gastroenteritis. These non-typhoidal serovars have very high estimated clinical incidences, causing deaths all over the world (38). Saleh et al. have shown that the proteome profiles of STM and SENT display differential expression of pathogenicity-associated genes as opposed to the typhoidal serovars (Typhi and Paratyphi A) (39). The problem of Salmonellosis is more pronounced with the emergence of AMR. These infections lead to substantial morbidity and mortality, particularly in regions with limited access to clean water, sanitation, and healthcare. WHO has estimated that *Salmonella* infections result in millions of cases and hundreds of thousands of deaths annually and has designated this pathogen in a high-priority group, highlighting the urgent need for effective therapeutic interventions (40).

IITR00803 appears as a promising broad-spectrum antibacterial agent with a better antibacterial profile against enteric pathogens, including *Salmonella* spp. and clinical isolates, with a favorable safety profile. Structurally, IITR00803 contains nitrothiophene along with a benzoxazole moiety. Benzoxazole derivatives are well-documented for their broad-spectrum antibacterial potential and diverse pharmacological properties. Several benzoxazole-based compounds are commercially available, serving as antibacterial agents (e.g., boxazomycin B, calcimycin) and muscle relaxants (e.g., chlorzoxazone) (41). Benzoxazole-based compounds are known to target bacterial DNA topoisomerases due to their structural similarity to pyrimidine bases—adenine and guanine (42).

In this study, IITR00803 demonstrated DNA-targeting activity specifically in *E. coli*, as evidenced by morphological changes upon treatment, whereas no such alteration was observed in other bacterial pathogens, including *Salmonella*. A detailed investigation of the species-specific mode of action of these small molecules can identify potential drug target(s) and can help in designing conditional therapeutic interventions. Furthermore, despite the absence of any modification in the nitrothiophene group, the small molecule exhibited no efflux susceptibility, which can be attributed to the Proton Motive Force (PMF) altering potential of the molecule, as RND efflux pumps are driven by PMF (43). In a study conducted by Hameed et al., the authors have designed derivatives of nitrothiophene carboxamides and demonstrated the changes in efflux susceptibility of the analogs without any alterations in the nitrothiophene moiety, but with the side group (8). This suggests that modifications in the distant parts of the chemical structure can alter the binding of molecules to efflux protein, which could also be the potential reason for IITR00803 to depict no efflux liability. Nonetheless, the unique structural framework of IITR00803 offers promising avenues to optimize this molecule for pharmacokinetic parameters and potential *in vivo* therapies to extend its clinical utility. The ability of IITR00803 to evade efflux pumps, coupled with its broad-spectrum antibacterial activity, lack of resistance acquisition, *in vivo* safety, and efficacy, positions it as a potential candidate for further development as a therapeutic option for treating infections caused by drug-resistant pathogens. Further research and optimization of IITR00803 could provide an innovative approach to addressing the urgent need for effective treatments against multidrug-resistant bacteria.

## MATERIALS AND METHODS

### Bacterial strains and plasmids

The bacterial strains used in this study are listed in Table S4. All strains were maintained on Muller Hilton (MH) Agar. Bacterial cells were grown in Muller Hilton Broth while conducting all the experiments until specified.

### Chemicals

A small molecule library was purchased from Maybridge (Now Thermo Fisher Scientific, USA). Phosphate buffer saline (PBS), H_2_DCF-DA, DiBAC_4_(5), and Sytox Orange probes were purchased from Thermo Fisher Scientific (USA). Phenylalanine-arginine-β-naphthylamide was procured from MedChemExpress, USA. Muller Hilton media, RPMI-1640 media, fetal bovine serum, and SS agar were purchased from Himedia, India. An *in situ* apoptosis detection kit was purchased from TaKaRa, India. All other chemical probes and antibiotics were purchased from Sigma-Aldrich (USA).

### MIC determination

MIC of the antibacterials was evaluated as per the CLSI guidelines (44). Twofold serial dilutions of antibacterial molecules were made in a 96-well plate in 100 µL MH broth. A 100 µL of 1:1,000 times diluted fresh bacterial culture (0.5 McFarland) prepared in MH broth was added to each well. Wells containing only MH broth were taken as the negative control, whereas wells with bacterial culture and without any antibacterial were taken as the positive control. The plate was incubated at 37°C under static conditions for 18 h. The highest concentration of antibacterial molecules with no visible bacterial growth was determined as the MIC. For overexpression strains of *nfsA*, *E. coli* AG1 pCA24N cells (wild type and overexpressor) were induced with 1 mM IPTG and then assessed for MIC (45).

### Checkerboard assay

A 2D checkerboard broth microdilution assay was conducted using twofold serially diluted concentrations of 2-nitrothiophene and benzoxazole in 96-well microplates in 100 µL MH broth (46). A 100 µL of 1:1,000 times diluted fresh bacterial culture (0.5 McFarland) of *E. coli* ATCC 25922 and *S. enterica* serovar Typhimurium cells prepared in MH broth were added to each well of their respective plate. Wells containing only MH broth were taken as the negative control, whereas wells with bacterial culture and without any antibacterial were taken as the positive control. The plate was incubated at 37°C under static conditions for 18 h. The highest concentration of antibacterial molecules with no visible bacterial growth was determined as the MIC. Type of chemical interaction was identified by calculating the fractional inhibitory concentration index (FICI) of the combination, which is defined as follows: FICI = (C_A_/MIC_A_) + (C_B_/MIC_B_), where MIC_A_ and MIC_B_ are the minimum inhibitory concentrations of antibiotics A and B, and C_A_ and C_B_ are the concentrations inhibiting bacterial growth in combination. Synergy is represented by FICI of ≤0.5, whereas antibiotics depict “no interaction” if FICI = 0.5–4, and antagonism is denoted by FICI values of >4 (47).

### Time-kill kinetics assay

The kill kinetics assay was performed as described previously with minor modifications (48). Briefly, *S. enterica* serovar Typhimurium cells (10^6^ CFU/mL) were treated with varying concentrations of IITR00803 for 24 h at 37°C, 180 rpm. Untreated cells and azithromycin-treated cells were taken as controls. Aliquots were withdrawn at regular intervals, serially diluted in 1× PBS, and spotted onto MH agar plates. The plates were incubated overnight at 37°C, and colony counts were determined as CFU/mL.

### *In vitro* post-antibiotic effect

*S. enterica* serovar Typhimurium cells (~10^6^ CFU/mL) were treated with IITR00803 for 1 h at 37°C, 180 rpm to assess the PAE of small molecules as described earlier with some modifications (10). Following the incubation, cells were washed with 1× PBS and resuspended in fresh MH broth. The cells were subsequently incubated further at 37°C, 180 rpm. Aliquots were withdrawn at regular intervals, serially diluted in 1× PBS, and spotted onto MH agar plates. The plates were incubated overnight at 37°C, and colony counts were determined as CFU/mL. Untreated cells and azithromycin (4 µg/mL) treated cells were taken as controls. The PAE was determined by the formula PAE = T – C, where T is the time taken for a unit Log_10_ increase in treated sample (CFU/mL) after drug removal, and C is the time taken for a unit Log_10_ increase (CFU/mL) in the untreated control.

### Biofilm inhibition assay

Anti-biofilm activity of IITR00803 was evaluated as described earlier with some modifications (49). For the biofilm eradication assay, an overnight-grown bacterial culture of *S. enterica* serovar Typhimurium was diluted 100 times in fresh 1/20 diluted tryptic soy MH broth. A total of 200 µL of the culture was added to each well of a 96-well plate. The plate was incubated at 37°C under static conditions for 72 h. Post-incubation, the media were removed, and fresh media containing different concentrations of IITR00803 were added. The plate was further incubated for 24 h at 37°C under static conditions. The wells were washed post-incubation with dH_2_O to remove the planktonic cells, followed by incubation at 45°C for 45 min to fix the biofilm. A 0.1% crystal violet (prepared in 1 [methanol]:3 [1× PBS]) was added to each well and incubated at room temperature for 20 min. The wells were washed twice with dH_2_O, and 75% methanol was added to each well. The absorbance at 590 nm was measured. For biofilm inhibition assay, an overnight-grown bacterial culture of *S. enterica* serovar Typhimurium was diluted 1000 times in fresh 1/20 diluted tryptic soy MH broth. A total of 100 µL of the culture was added to each well of a 96-well plate containing drug dilutions in 100 µL broth. The plate was incubated at 37°C under static conditions for 72 h. Post-incubation, biofilm was estimated as described above. Untreated cells and azithromycin-treated cells were used as controls for the biofilm formation assay.

### Reactive oxygen species generation assay

*S. enterica* serovar Typhimurium and *E. coli* BW25113 cultures (O.D._600_ ~0.3) were treated with different concentrations of IITR00803 (4, 8, and 16 µg/mL) for 1 h at 37°C, 180 rpm in MH broth for ROS detection as described previously with some modifications (16). The cells were washed with 1× PBS after incubation, and the cells were resuspended in 1× PBS containing 100 µM 2′,7′-dichlorodihydofluorescein diacetate (H_2_DCF-DA) dye and incubated at 37°C for 1 h. The absorbance at 600 nm and fluorescence at excitation and emission wavelengths of 485 and 528 nm, respectively, were measured post-incubation using a Biotek Synergy H1 plate reader. Relative fluorescence units (RFU) were calculated by normalizing the fluorescence values by the respective absorbance values. H_2_O_2_-treated cells were used as a positive control.

### Membrane potential assay

*S. enterica* serovar Typhimurium and *E. coli* BW25113 cultures (O.D._600_ ~0.3) were treated with different concentrations of IITR00803 for 1 h at 37°C, 180 rpm in MH broth for membrane potential assessment as described earlier with some modifications (18). The cells were washed with 1× PBS after incubation, and the cells were resuspended in 1× PBS containing 5 µg/mL DiBAC_4_(5) dye and incubated at 37°C for 1 h. The absorbance at 600 nm and fluorescence at excitation and emission wavelengths of 485 and 525 nm, respectively, were measured post-incubation using a Biotek Synergy H1 plate reader. RFUs were calculated by normalizing the fluorescence values by the respective absorbance values. CCCP-treated cells were used as a positive control.

### NAD(P)H estimation assay

*S. enterica* serovar Typhimurium and *E. coli* BW25113 cultures (O.D._600_ ~0.3) were treated with different concentrations of IITR00803 for 2 h at 37°C, 180 rpm in MH broth for NAD(P)H estimation as described earlier with some modifications (50). The cells were washed with 1× PBS after incubation and resuspended in 1× PBS containing 1 µg/mL resazurin and incubated at 37°C for 2 h. The absorbance at 600 nm and fluorescence (resorufin) at excitation and emission wavelengths of 550 and 590 nm, respectively, were measured post-incubation using a Biotek Synergy H1 plate reader. RFUs were calculated by normalizing the fluorescence values by the respective absorbance values. CCCP-treated cells were used as a positive control.

### Antibiotic potentiation

Twofold serial dilutions of antibiotics were made in a 96-well plate in 100 µL MH broth. 100 µL of 1:1,000 times diluted fresh *S. enterica* serovar Typhimurium culture (0.5 McFarland) prepared in MH broth was added to each well. Two variations of these plates were prepared such that one plate contained only antibiotic dilutions and another one contained antibiotic dilutions along with 2 µg/mL of IITR00803 (0.5× MIC), respectively. The plates were incubated at 37°C under static conditions for 18 h. The highest concentration of antibiotic in the presence or absence of IITR00803 with no visible bacterial growth was determined as the MIC of antibiotic in the specific condition.

### Membrane damage assay

*S. enterica* serovar Typhimurium and *E. coli* BW25113 cultures (O.D._600_ ~0.3) were treated with different concentrations of IITR00803 for 1 h at 37°C, 180 rpm in MH broth for membrane damage evaluation as described earlier with some modifications (51). The cells were washed with 1× PBS after incubation, and the cells were resuspended in 1× PBS containing 1 µM Sytox Orange dye and incubated at 37°C for 1 h. The absorbance at 600 nm and fluorescence at excitation and emission wavelengths of 547 and 570 nm, respectively, were measured post-incubation using a Biotek Synergy H1 plate reader. RFUs were calculated by normalizing the fluorescence values by the respective absorbance values. Polymyxin B-treated cells were used as a positive control.

### Scanning electron microscopy

Freshly prepared *S. enterica* serovar Typhimurium culture of O.D._600_ ~0.3–0.4 was taken and treated with varying concentrations of IITR00803 (8 and 16 µg/mL) for 2 h at 37°C, 180 rpm in MH broth as described previously with some modifications (52). After incubation, cells were washed with 1× PBS and fixed with 2% formaldehyde prepared in 1× PBS. The samples were incubated at 4°C for 2 h, followed by washing with 1× PBS. The samples were finally resuspended in 1× PBS, and 10 µL of each sample was dropped on a clean cut glass slide and allowed to dry. Dried samples were sequentially dehydrated using 30%, 50%, 70%, 90%, and 100% ethanol for 15 min each. The samples were gold-coated before being visualized under a scanning electron microscope.

### Dual fluorescent protein reporter assay

*E. coli* BW25113 Δ*tolC* carrying pDualRep2 plasmid culture of O.D._600_ ~0.2 was treated with varying concentrations of IITR00803 (16, 32, and 64 µg/mL) for 4 h at 37°C, 180 rpm in MH broth (23). After incubation, O.D._600_ and fluorescence readings at excitation and emission wavelengths of 532 and 588 nm, respectively, were measured using a Biotek Synergy H1 instrument. RFUs were calculated by normalizing the fluorescence values by the respective absorbance values. Ciprofloxacin-treated cells were used as a positive control. For imaging of pDualrep2-induced bacterial cells, agar plates covered with *E. coli* DH5α strain transformed with pDualrep2 plasmid were spotted with IITR00803, levofloxacin (DNA-damaging antibiotic), and erythromycin (protein synthesis inhibitor). The plate was scanned in the Cy3 and Cy5 channels to detect RFP and Katushka2S fluorescence. The images were captured using a Bio-Rad ChemiDoc Imaging System. Pseudo-green and red colors were added to show *sulA* and *katushka2S* induction, respectively.

### TUNEL assay

The TUNEL assay was performed as described earlier with some modifications using the Takara Apoptosis detection kit as per the manufacturer’s protocol (53). *E. coli* BW25113 Δ*tolC* culture of O.D._600_ ~0.3 was treated with 5× MIC of IITR00803 (40 µg/mL) for 2 h at 37°C, 180 rpm. After treatment, cells were fixed with 4% formaldehyde prepared in 1× PBS and incubated on ice for 30 min. Following incubation, the samples were washed with 1× PBS and resuspended in 70% ice-cold ethanol. The resuspended samples were stored at −20°C for 48 h. The samples were washed with 1× PBS and resuspended in 100 µL permeabilization buffer on ice for 5 min. The cells were washed with 1× PBS, and the pellet was resuspended in 50 µL labeling mix with incubation at 37°C for 90 min. After incubation, samples were washed and resuspended in 1× PBS for fluorescence-activated cell sorting (FACS) analysis. The FITC signal was analyzed with an excitation laser at 488 nm and a band pass filter of 525/15 nm using a BD flow cytometer (BD FACSLyric), and 10,000 events were captured. Half offset histograms were created using FlowJo software, representing a minimum of 97% population. Ciprofloxacin-treated cells were used as a positive control.

### Resistant mutant generation

The resistant mutant generation assay was performed as described earlier with some modifications (54). *S. enterica* serotype Typhimurium cells were exposed to sub-inhibitory concentrations of IITR00803 with an incubation at 37°C, 18 h for a period of 36 days. The cells that survived the highest concentration of IITR00803 were assessed for their MIC after passaging them in a drug-free medium at the end of 36 days to evaluate the stability of the resistant mutant generated.

### Hemolysis assay

Hemolysis assay was performed as described previously with some modifications (55). Freshly isolated 1 mL blood was taken and washed with 1× PBS by centrifugation at 2,000 rpm for 10 min. The blood was diluted 125 times in 1× PBS. Diluted blood was treated with an equal volume of 1× PBS containing varying concentrations of IITR00803 in a 96-well plate. The plate was incubated for 1 h at 37°C under static conditions. Triton X-100 (0.1%) treatment was used as a positive control. After incubation, the samples were centrifuged at 2,000 rpm for 10 min. The supernatant was collected in a fresh 96-well plate, and absorbance was measured at 540 nm. Percent hemolysis was calculated by using the following formula:


$$Percent\;hemolysis=\left(\frac{Absorbance\;(sample)-Absorbance\;(untreated\;control)}{Absorbance\;(positive\;control)-Absorbance\;(untreated\;control)}\right)\;\times 100.$$

### *In vitro* toxicity assay

Freshly isolated blood (10 mL) was used for the isolation of PBMCs and PMNs using Polymorphprep density gradient media (Serumwerk Bernburg AG, Germany) as per the manufacturer’s instructions. *In vitro* toxicity assessment was performed as described earlier with some modifications (56). Briefly, 10^4^ cells resuspended in RPMI (Roswell Park Memorial Institute) 1640 medium supplemented with 10% fetal bovine serum were seeded in each well of the cell culture 96-well plate and incubated at 37°C with 5% CO_2_ for 24 h. The spent media was removed from the wells after 24 h, and fresh media containing varied concentrations of IITR00803 were added to the cells and incubated for 24 h at 37°C with 5% CO_2_. After incubation, the media was removed from the wells and 100 µL of 0.5 mg/mL MTT solution was added to each well and incubated at 37°C with 5% CO_2_ for 4 h. Subsequently, MTT was removed and 100 µL of DMSO was added to the wells, and the plate was incubated at 37°C for 10 min. The absorbance at 570 nm was measured, and percent survival was calculated by using the following formula:


$$Percent\;survival=\left(\frac{Absorbance\;(sample)-Absorbance\;(untreated\;control)}{Absorbance\;(positive\;control)-Absorbance\;(untreated\;control)}\right)\;\times 100.$$

### *In vivo* toxicity assessment in the *C. elegans* model

*In vivo* toxicity study of IITR00803 was performed in a temperature-sensitive *C. elegans* strain AU37 to maintain sterility of worms throughout the experiment as described earlier with slight modifications (57). Synchronized L2 stage worms (*n* = 155–175) were taken in M9 buffer supplemented with 1 mM MgSO_4_ and *E. coli* OP50 and treated with 10× and 20× MICs of IITR00803 corresponding to 40 and 80 µg/mL. Sodium fluoride (100 µg/mL) treatment was used as a positive control. The live worms were counted every day for 5 days using an Axioscope A1 fluorescence microscope equipped with an AxioCam MRC digital camera (Zeiss, Germany). The worms were maintained at 25°C throughout the experiment to maintain their sterility.

### *In vivo* efficacy study in the *C. elegans* model

*In vivo* efficacy study of IITR00803 was performed on the *C. elegans* model infected with *S. enterica* serovar Typhimurium. Egg preparation from a plate of *C. elegans* AU37 grown on NGM agar supplemented with *E. coli* OP50 as food was done by bleach and potassium hydroxide (58). Eggs were transferred to a fresh NGM plate containing *S. enterica* serovar Typhimurium for infection. After 48 h, the worm suspension was treated with 30 µg/mL of gentamicin for 30 min, followed by washing with M9 buffer. The worms were resuspended in M9 buffer supplemented with 1 mM MgSO_4_. A 100 µL of worm suspension was aliquoted in a 96-well plate (a minimum of 160 worms, inclusive of four replicates, was taken for each sample) and treated with 5× and 10× MICs of IITR00803 (20 and 40 µg/mL) for 12 h. Azithromycin (5 and 10 µg/mL) treatment was taken as positive control. Worms were disrupted with sterilized silicon carbide particles post-treatment, and samples were serially diluted in 1× PBS and plated on an SS agar plate (Himedia, India). The plates were incubated at 37°C overnight, and colonies were counted.

## Data Availability

All data generated or analyzed during this study are included in this published article and its supplementary information files.

## SUPPLEMENTAL MATERIAL

Supplemental figures and tables spectrum.01144-25-s0001.docx Fig. S1 to S3 and Tables S1 to S4.

ASM does not own the copyrights to Supplemental Material that may be linked to, or accessed through, an article. The authors have granted ASM a non-exclusive, world-wide license to publish the Supplemental Material files. Please contact the corresponding author directly for reuse.
