# Supplementary material for: IITR00803: a benzoxazole-nitrothiophene small molecule with broad-spectrum antibacterial potential
Source: Microbiol Spectr. 2025 Aug 12;13(9):e01144-25. doi: 10.1128/spectrum.01144-25 (PMC12403614; doi:10.1128/spectrum.01144-25)
Supplement: Supplemental figures and tables — Fig. S1 to S3 and Tables S1 to S4. [file spectrum.01144-25-s0001.docx]

**Supplementary Information**

**IITR00803: A Benzoxazole-Nitrothiophene Small Molecule with Broad-spectrum antibacterial potential**

Rinki Gupta^a^, Amit Gaurav^a^, and Ranjana Pathania^a,*^

^a^Department of Biosciences and Bioengineering, Indian Institute of Technology Roorkee, Roorkee, Uttarakhand 247 667, India

*Address correspondence to Ranjana Pathania, ranjana.pathania@bt.iitr.ac.in

**Running Title:** Exploring the antibacterial properties of IITR00803

**Table S1:** Minimum inhibitory concentrations (MICs) of IITR00803, 2-nitrothiophene and benzoxazole.

| Bacterial strain | MIC (µg/mL) | | |
| --- | --- | --- | --- |
|  | **IITR00803** | **2-nitrothiophene** | **Benzoxazole** |
| *E. coli* ATCC 25922 | 16 | >128 | >128 |
| *S. enterica* serovar Typhimurium | 4 | 64 | >128 |

**Table S2:** Minimum inhibitory concentrations (MICs) of IITR00803 against *E. coli* AG1 pCA24N and pCA24N::*nfsA* strains induced by 1 mM IPTG.

| Bacterial strain | MIC (µg/mL) |
| --- | --- |
|  | **IITR00803** |
| *E. coli* AG1 pCA24N | 4 |
| *E. coli* AG1 pCA24N::*nfsA* | 4 |

**Table S3:** Fractional inhibitory concentration index (FICI) of IITR00803 and aminoglycoside antibiotic combinations. ND denotes not determined.

| Aminoglycoside | FICI | |
| --- | --- | --- |
|  | *S. enterica* serovar Typhimurium | *E. coli* |
| Tobramycin | 0.75 | ND |
| Amikacin | 0.53 | 0.5625 |
| Kanamycin | 0.75 | 0.375 |
| Spectinomycin | 0.5 | 0.5 |
| Hygromycin | 0.5 | ND |
| Apramycin | 0.5 | ND |
| Streptomycin | 0.75 | ND |
| Gentamicin | 0.56 | 0.375 |

**Table S4:** List of strains used in the study.

| Bacterial strain/ plasmid | Source |
| --- | --- |
| *E. coli* ATCC 25922 | ATCC, USA |
| *E. coli* MC1061 | Lab collection |
| *E. coli* AG1 pCA24N | ASKA collection |
| *E. coli* AG1 pCA24N::*nfsA* | ASKA collection |
| *E. coli* BW25113 | Lab collection |
| *E. coli* BW25113 Δ*tolC* | Keio collection |
| *E. coli* O157:H7 | Lab collection |
| *Shigella flexneri* ATCC 9199 | ATCC, USA |
| *Salmonella enterica* serovar Choleraesuis ATCC 10708 | ATCC, USA |
| *S. enterica* serovar Typhimurium MTCC 98 | MTCC, India |
| *S. enterica* serovar Enteritidis (NKN177) | Lab collection |
| *Vibrio fluvialis* (BD-146) | Lab collection |
| *V. fluvialis* (L-15318) | Lab collection |
| *Acinetobacter baumannii* ATCC 17978 | ATCC, USA |
| *A. baumannii* AYE | Lab collection |
| *Klebsiella pneumoniae* ATCC 700603 | ATCC, USA |
| *Pseudomonas aeruginosa* MTCC 2453 | MTCC, India |
| *Burkholderia cepacia* ATCC 25416 | ATCC, USA |
| *Staphylococcus aureus* ATCC 29213 | ATCC, USA |
| *Bacillus cereus* ATCC 11778 | ATCC, USA |
| *Listeria monocytogenes* NKN8 | Lab collection |
| *Mycobacterium smegmatis* RPT45 | Lab collection |
| *Enterococcus faecium* NKN18 | Lab collection |
| *Enterococcus faecalis* ATCC 29212 | Dr. Rajni Gaind, VMMC, India |
| *E. coli* BW25113 Δ*tolC* | Keio collection |
| *E. coli* BW25113 Δ*acrA* | Keio collection |
| *E. coli* BW25113 Δ*acrB* | Keio collection |
| *E. coli* BW25113 Δ*mdfA* | Keio collection |
| *E. coli* BW25113 Δ*mdtA* | Keio collection |
| *E. coli* BW25113 Δ*ompF* | Keio collection |
| *E. coli* BW25113 Δ*emrD* | Keio collection |
| *E. coli* BW25113 Δ*nfsA* | Keio collection |
| *E. coli* BW25113 Δ*nfsB* | Keio collection |
| *S. enterica* serovar Typhi Vi positive | Lab collection |
| NASF Sal 1-24 (24 poultry isolates of Salmonella) | Lab collection |
| RPT Sal 1 (GMCH-14) | Dr. Varsha Gupta, GMCH, India |
| RPT Sal 2 (GMCH-15) | Dr. Varsha Gupta, GMCH, India |
| *S. flexneri* RPTS strains | Lab collection |
| *Vibrio cholerae* NKN43 | Lab collection |
| pDualRep2 (Plasmid) | Lab collection |

**Figure S1:** Bar graph representing biofilm inhibition of *S. enterica* serovar Typhimurium in the presence of different concentrations of IITR00803. Azithromycin was used as positive control. Data (n=3) are represented as Mean ± S.D, *p* values were determined by one-way ANOVA followed by Dunnett’s multiple comparison test. ***, *p*<0.001; *, *p*<0.05.


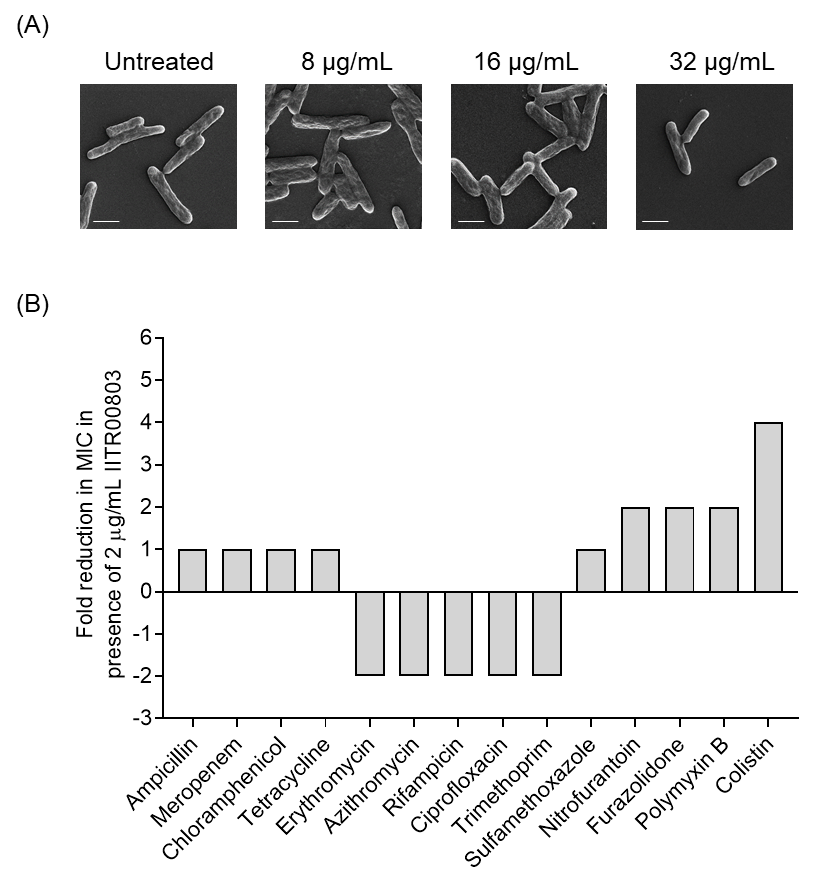


**Figure S2:** (A) Scanning electron micrographs of *S. enterica* serovar Typhimurium cells upon treatment with IITR00803. Scale bars represent 1 µm. (B) Bar graph representing fold change in MICs of antibiotics in the presence of 2 µg/mL IITR00803 (0.5X MIC) in *S. enterica* serovar Typhimurium.


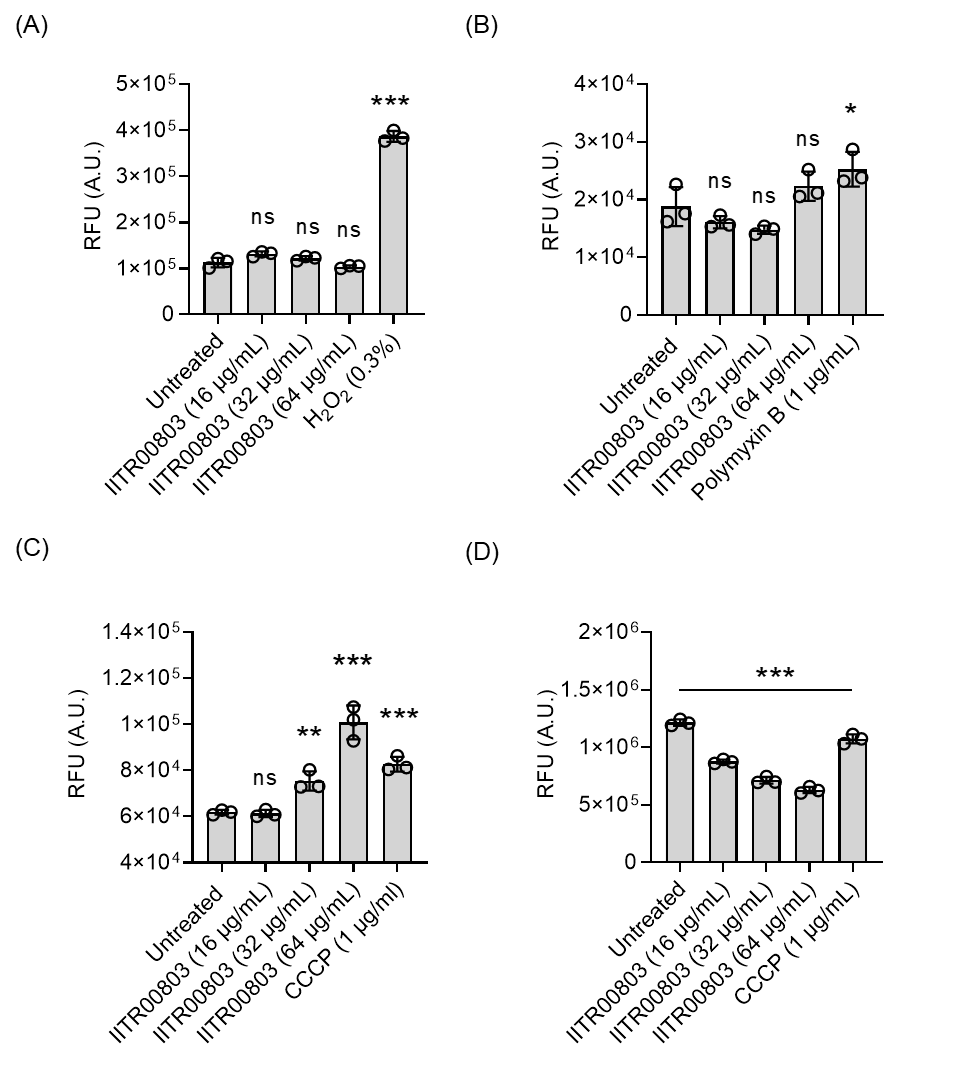


**Figure S3:** IITR00803 alters cellular energetics in *E. coli*. (A) Relative fluorescence of H_2_DCF-DA upon IITR00803 treatment. H_2_O_2_ was used as a positive control. (B) Relative fluorescence of Sytox Orange^TM^ upon IITR00803 treatment. Polymyxin B was used as a positive control. (C) Relative fluorescence of DiBAC_4_ upon IITR00803 treatment. CCCP was used as a positive control. (D) Relative fluorescence of resazurin showing NAD(P)H level after IITR00803 treatment. CCCP was used as a positive control. Data (n=3) are represented as Mean ± S.D., *p* values were determined by one-way ANOVA followed by Dunnett’s multiple comparison test. *, *p*<0.05; **, *p*<0.01; ***, *p*<0.001; ns represents non-significant.
